# Metabolic Consequences of Infection of Grapevine (*Vitis vinifera* L.) cv. “Modra frankinja” with Flavescence Dorée Phytoplasma

**DOI:** 10.3389/fpls.2016.00711

**Published:** 2016-05-23

**Authors:** Nina Prezelj, Elizabeth Covington, Thomas Roitsch, Kristina Gruden, Lena Fragner, Wolfram Weckwerth, Marko Chersicola, Maja Vodopivec, Marina Dermastia

**Affiliations:** ^1^Department of Biotechnology and Systems Biology, National Institute of BiologyLjubljana, Slovenia; ^2^Department of Plant and Environmental Sciences, Copenhagen Plant Science Centre, University of CopenhagenTaastrup, Denmark; ^3^Global Change Research Centre, Czech Globe AS CR, v.v.i.Drásov, Czech Republic; ^4^Department of Ecogenomics and Systems Biology, Faculty of Life Sciences, University of ViennaVienna, Austria; ^5^Vienna Metabolomics Center (VIME), University of ViennaVienna, Austria; ^6^Jožef Stefan International Postgraduate SchoolLjubljana, Slovenia

**Keywords:** fructose, gene expression, metabolome, starch, sucrose synthase, SWEET17a

## Abstract

Flavescence dorée, caused by the quarantine phytoplasma FDp, represents the most devastating of the grapevine yellows diseases in Europe. In an integrated study we have explored the FDp–grapevine interaction in infected grapevines of cv. “Modra frankinja” under natural conditions in the vineyard. In FDp-infected leaf vein-enriched tissues, the seasonal transcriptional profiles of 14 genes selected from various metabolic pathways showed an FDp-specific plant response compared to other grapevine yellows and uncovered a new association of the SWEET17a vacuolar transporter of fructose with pathogens. Non-targeted metabolome analysis from leaf vein-enriched tissues identified 22 significantly changed compounds with increased levels during infection. Several metabolites corroborated the gene expression study. Detailed investigation of the dynamics of carbohydrate metabolism revealed significant accumulation of sucrose and starch in the mesophyll of FDp-infected leaves, as well as significant up-regulation of genes involved in their biosynthesis. In addition, infected leaves had high activities of ADP-glucose pyrophosphorylase and, more significantly, sucrose synthase. The data support the conclusion that FDp infection inhibits phloem transport, resulting in accumulation of carbohydrates and secondary metabolites that provoke a source-sink transition and defense response status.

## Introduction

Phytoplasma are cell-wall-free plant pathogenic bacteria that form a monophyletic clade within the class Mollicutes (Maejima et al., 2014; Marcone, 2014; Zhao et al., 2015). Both their cell and genome size are the smallest among the bacteria. In plants, phytoplasma exclusively inhabit nutrient-rich phloem tissues. They can be transmitted from plant to plant by sap-feeding insect vectors of the order Hemiptera, and they propagate within the cytoplasm of both these insects and their plant hosts. However, phytoplasmas are still the most poorly characterized plant pathogens because of their low concentration in plants, because of unsuccessful efforts at gene delivery and mutagenesis, and because they have resisted attempts at routine cultivation *in vitro*. Recent complete sequencing of four phytoplasma genomes and their comparative analysis have increased our understanding of phytoplasma genetics and revealed that these plant pathogens experienced substantial evolutionary gene decay, gene loss, and disruption or loss of important biosynthetic pathways (Kube et al., 2012). These genomic changes may account for their complete reliance on the host plant or insect cells for survival.

Phytoplasmas have a broad range of plant hosts among the monocots and dicots, and diseases of many important crops are associated with these pathogens. At least 10 phytoplasma ribosomal subgroups have been associated with grapevine yellows diseases (Constable, 2010), which have great economic impact on viticulture. Different grapevine-infecting phytoplasma cause nearly identical symptoms of hard and brittle texture of leaves, leaf curling and discoloration of leaf veins and laminas, interveinal yellowing or reddening, uneven or total lack of lignification of canes, flower abortion, and berry withering (Hren et al., 2009a; Prezelj et al., 2013). Because the symptoms are indistinguishable by visual inspection they are assumed to be caused by similar mechanisms. However, the epidemiology associated with specific phytoplasma species differs (Constable, 2010). In Europe, the main phytoplasma associated with grapevine yellows are the causal agent of flavescence dorée (henceforth: FDp) and “*Candidatus* Phytoplasma solani” (henceforth: BNp), which causes bois noir (Quaglino et al., 2013). Based on 16S rRNA sequence similarities these causal agents belong to the 16SrV (Lee et al., 2004) and 16SrXII-A (Lee et al., 2007) ribosomal groups, respectively. In terms of economic impact, FDp is the most severe and most dangerous of these two. FDp has been recorded in French vineyards since the mid-1950s (Boudon-Padieu, 2002), and it is rapidly spreading in a northeast direction to other countries (Bertaccini, 2014) probably first by the transport of contaminated plant material and then by natural transmission via the non-indigenous monophagous leafhopper *Scaphoideus titanus* (Arnaud et al., 2007; Papura et al., 2009, 2012; Malembic-Maher et al., 2011). In the absence of appropriate control measures, the number of infected grapevines is likely to increase by up to 40-fold every year (Prezelj et al., 2013), to reach 80–100% infection rates within a few years (Steffeck et al., 2007). Due to its epidemic potential, FDp is listed in the EU2000/29 Council Directive on Harmful Organisms and the EPPO A2 quarantine list of pests, and the uprooting, and destruction of diseased plants is mandatory.

The quarantine status of FDp and its consequences, together with current inability to cultivate FDp under cell-free conditions, limit studies on the underlying molecular mechanisms involved in the interactions between grapevines and FDp. However, some recent studies on plant responses to FDp infection have focused on the late growing season, when the symptoms of disease are clearly visible, to analyze the transcriptome and targeted gene expression, as well as the proteome of infected plants or plants that have recovered from infection (Margaria and Palmano, 2011; Margaria et al., 2013; Gambino et al., 2013). These studies exposed differential regulation of proteins involved in photosynthesis, pathogenesis-related proteins (PRP) from class 5 and proteins related to antioxidant responses. The results corroborate outcomes from similar studies on grapevines infected with BNp (Hren et al., 2009a,b; Landi and Romanazzi, 2011; Santi et al., 2013a,b).

Growing evidence has indicated that pathogens, including phytoplasmas infecting *Catharanthus roseus*, coconut palm, maize, and *Vicia faba*, can affect the carbohydrate metabolism of host plants (Lepka et al., 1999; Maust et al., 2003; Junqueira et al., 2004; Hren et al., 2009a,b; Santi et al., 2013b; Gai et al., 2014; Rojas et al., 2014) possibly by inhibiting phloem transport. Callose depositions have been observed in the sieve elements of *C. roseus, Euphorbia pulcherrina* (Christensen et al., 2004), and *V. faba* infected with FDp (Musetti et al., 2013). The latter has been shown to be the result of Ca^2+^ influx into sieve tubes. Sieve plate callose occlusion is in agreement with significant induction of callose synthase transcript in grapevine infected with BNp as well as induction of transcripts of enzymes for sucrose cleavage (Hren et al., 2009a; Santi et al., 2013b) and accumulation of soluble sugars, sucrose and starch in the source infected leaves (Lepka et al., 1999; Maust et al., 2003; Gai et al., 2014). Based on these observations it has been suggested that phytoplasmas induce a switch from carbohydrate source to sink in the leaves of infected plants. Changes in photosynthate translocation along with other impaired physiological functions, including reduced photosynthesis, stomatal conductance, hydrogen peroxide accumulation, altered secondary metabolism, and disturbed plant hormone balance, could account for symptoms exhibited by phytoplasma infected *C. roseus* (Lepka et al., 1999; Choi et al., 2004), tobacco (Lepka et al., 1999), coconut palm (Maust et al., 2003), *E. pulcherrima* (Nicolaisen and Horvath, 2008), and grapevine (Bertamini and Nedunchezhian, 2001; Musetti et al., 2007; Rusjan et al., 2012a,b; Gambino et al., 2013; Vitali et al., 2013; Margaria et al., 2014; Rusjan and Mikulič-Petkovšek, 2015). Although, transcripts of the genes that encode the enzymes involved in the production of carbohydrates have been shown to be increased in BNp-infected plants (Hren et al., 2009a,b; Landi and Romanazzi, 2011; Santi et al., 2013a,b), they have not been examined in detail in FDp-infected grapevines. Moreover, the carbohydrate metabolites themselves and their corresponding enzymes have not been analyzed in phytoplasma-infected grapevines.

Recently, a novel family of hexose and sucrose transporters was discovered in animals and plants and named SWEETs (Eom et al., 2015). The SWEETs appear to function as bidirectional uniporters that facilitate the diffusion of hexose and sucrose across cell membranes. Their function is, however, also targeted by plant bacterial and fungal pathogens, which have been shown to alter the expression of the *Arabidopsis* and rice *SWEET* genes and to modulate this sugar transport system to their own advantage (Chen et al., 2010). In addition, in response to *SAP11* in an *Arabidopsis* transgenic line, the levels of *SWEET1* transcript were significantly increased (Lu et al., 2014). SAP11 is an effector of the Witches' Broom strain of aster yellows phytoplasma, which in *Arabidopsis* triggers phosphorus starvation responses and suppresses plant defense against bacterial pathogens (Lu et al., 2014). Sixteen *SWEET* homologs have been predicted for the grapevine genome, although none of these has yet been studied in detail (Lecourieux et al., 2014). Accordingly, there are no reports available about connections between grapevine SWEETs and pathogenesis.

Although metabolites are the end products of cellular regulatory processes, and their levels can be regarded as the ultimate responses of biological systems to genetic or environmental changes (Fiehn, 2002; Weckwerth, 2011), few metabolomic data from phytoplasma-infected plants are available (Choi et al., 2004; Gai et al., 2014), and none of these relate to grapevine.

This first integrated study of spatial and temporal dynamics of FDp-host grapevine interactions includes transcriptional and metabolomic analysis of leaf vein-enriched tissue and biochemical analysis of whole leaves. For several important reasons this study was carried out in a production vineyard of cv. “Modra frankinja” (syn. “Blaufränkisch”) with confirmed FDp infection. Specifically, (i) while studies of plant responses to pathogen infection conducted under controlled laboratory or glasshouse conditions can focus on the impact of individual factors, it is the resulting changes in the analyzed parameter profiles of field-established systems that can uncover the complexity of the signaling networks involved in pathogenicity (Schmidt et al., 2004; Izawa, 2015). (ii) Cultivation of phytoplasma in axenic cultures (Contaldo et al., 2012) is still far from being a routine procedure. (iii) Grafting of infected grapevine tissues from the vineyard onto container-grown grapevines as an alternative to axenic culturing has limited applicability, as the percentage of successfully transmitted FDp is only 1.4% due to the high recovery rate under glasshouse conditions (data not shown). At the same time, FDp-infected plant material in vineyards can also be very limited. To avoid regulatory requirements to destroy diseased plants, we maintained plants in the vineyard under a quarantine net, allowing us to follow the plants over two consecutive years.

We specifically compared five aspects of uninfected and FDp-infected grapevines. (i) The expression patterns of several genes that had previously been shown to be associated with the symptomatic phase of infection by the taxonomically unrelated phytoplasma BNp (Hren et al., 2009a) were investigated in leaf vein-enriched tissues, to determine whether the similar symptoms of flavescence dorée and bois noir are associated with a more general plant response to phytoplasma that cause grapevine yellows diseases. Here, the gene expression patterns in spring before symptom development were also examined, to investigate any early markers of flavescence dorée. (ii) The gene expression changes of some SWEET family genes in FDp-infected leaf vein-enriched samples were evaluated. (iii) The leaf vein-enriched metabolomes of uninfected and FDp-infected grapevines were investigated. (iv) Several of the carbohydrates in whole leaves of uninfected and FDp-infected grapevines were examined. (v) The signatures of the key enzymes involved in metabolic conversions of the carbohydrates were determined. To follow the activities of these enzymes, an experimental platform that was established for herbaceous plants (Jammer et al., 2015) has been adopted and optimized for woody grapevines.

## Materials and methods

The work-flow for the experimental procedures is illustrated in Supplementary Figure S1.

### Locations and sampling

This study of FDp-infected grapevines (*Vitis vinifera* L.) was carried out in a production vineyard of cv. “Modra frankinja” (syn. “Blaufränkisch”). The vineyard was located in south-eastern Slovenia (45°47′ N, 15°4′ E), under a continental climate with snowy winters and hot summers. Before the uprooting and destruction of several FDp-infected grapevines in the vineyard in 2010, a short row was selected that included four plants with confirmed presence of FDp by qPCR in July 2010 (henceforth: infected); and 11 plants in which FDp was not detected by qPCR (henceforth: uninfected). This entire row was protected with a quarantine net, which allowed us to avoid the mandatory uprooting of these infected grapevines and thus allowed their continued growth over the subsequent season. The use of the net was approved by the Phytosanitary Administration of the Republic of Slovenia. The grapevines inside the net were subjected to the same antifungal treatments and pruning as the grapevines outside the net, and they were also treated against the FDp vector *Scaphoideus titanus*. In 2011 FDp was confirmed by qPCR in all symptomatic plants in June, July and August.

Our six-year-long transcriptional study and subsequent statistical analysis of grapevine infected with BNp together with several environmental factors has shown that phytoplasma infection has a dominant influence on the transcriptional profile compared to environmental factors. Among the environmental factors only summer rainfall was found to be significantly associated with symptom development and thus also influenced the intensity of physiological response. However, rainfall more heavily affects recovered and newly infected plants and has only a slight effect on already infected plants (Rotter et al., in review). Due to lack of experimental material because of the quarantine status of FDp, the two-year results from the present study, which comprised four plants with established FDp infection and six uninfected plants (Supplementary Table S1), were compared based on these assumptions. The sampling for carbohydrate, metabolome, and enzyme analysis was carried out in summer 2010, and for gene expression analysis through the entire growing season of 2011. For all of the analyses, uninfected leaves (i.e., with no visual symptoms and no phytoplasma detected by qPCR) and infected leaves were sampled. To ensure as much as possible comparable conditions each sampling of fully developed leaves from approximately the third to the fifth leaf from the end of the shoots, and 1 to 2 m above the ground on the sunny side of the grapevines was performed in a single day, during the light period between 10:00 and 13:00 h (Hren et al., 2009a). For the gene expression and metabolome studies, three main veins of uninfected and infected leaf were cut out using a sterile scalpel, and for determination of carbohydrates and enzyme activities, whole leaves were sampled. All of the samples were immediately frozen in the field in liquid nitrogen and stored at –80°C until homogenization.

### Grapevine sanitary status determination

Each grapevine included in the study was tested for FDp and BNp according to the detection system developed by Hren et al. (2007). DNA extraction was performed from leaf veins as reported by Prezelj et al. (2013). Testing revealed no BNp in the selected FDp-infected plants. A parallel molecular study of the FDp isolates involved in this analysis showed that all of the sampled grapevines were infected with isolates belonging to the 16SrV-D subgroup (Mehle et al., 2011). All of the sampled grapevines were also tested for viruses, using double antibody sandwich enzyme-linked immunosorbent assay (ELISA) with antibodies against *Grapevine fanleaf virus, Grapevine virus A, Grapevine leafroll associated virus* (GLRaV)-1, GLRaV-2, GLRaV-6, and *Grapevine fleckvirus* (GFkV) (Bioreba), and plate trapped antigen ELISA for *Grapevine virus B* (Agritest), according to the producer's instructions. The optical densities of all of the samples were measured at 405 nm; the ELISA reads were considered positive when these reached >2-fold of the negative controls. In further analysis, only grapevines that were virus-free and the one infected with GFkV were included. In grapevines, GFkV is generally latent and asymptomatic (Sabanadzovic et al., 2000), as has also been shown in a study of “Chardonnay” grapevines infected with BNp (Hren et al., 2009a). The sanitary status for each of the individual grapevines is shown in Supplementary Table S1.

### Bioinformatics analysis

To search for SWEET family proteins in the grapevine genome, the PSI-BLAST algorithm was used to compare the *Arabidopsis thaliana* and *Oryza sativa* SWEET protein sequences from GenBank against the 12 × coverage proteome data of *V. vinifera* (Pinot noir PN40024) generated by Genoscope, the Institute of Applied Genomics, and the University of Verona (Jaillon et al., 2007) and the 7 × coverage genome data released by the Agraria Institute of San Michele all'Adige (Velasco et al., 2007). Candidate sequences were aligned using ClustalX2 and a neighbor-joining phylogram was constructed, with both using the default parameters. The NCBI Conserved Domain Search tool was used to examine the sequences for conserved regions against the Conserved Domains Database, v3.13 (Derbyshire et al., 2015).

### Gene expression analysis

The final list of genes for which expression was examined is given in Table 1, along with their protein functions, and assay-related information is given in Table 2.

**Table 1:** **Genes and their functional groups and protein products targeted in this study**.

**Functional target**	**Gene**	**Protein function**	**Protein**
Carbohydrate metabolism	*VvSUSY4*	Sucrose synthase	NDP-glucose:D-fructose 2-alpha-D-glucosyltransferase
	*VvINV2*	Vacuolar acid invertase	Acid β-fructofuranosidase
	*VvAGPL*	Large subunit of ADP-glucose pyrophosphorylase	Glucose-1-phosphate adenylyltransferase
	*VvSWEET1*	Glucoside transporter	SWEET1 homolog in *Arabidopsis* is a glucose transporter
	*VvSWEET10*	Putative sucrose transporter	SWEET10
	*VvSWEET17a*	Putative tonoplast fructose transporter	SWEET17a
Antioxidants	*VvAPX6*	Antioxidant enzyme	L-Ascorbate peroxidase 6
	*VvGPX4*	Antioxidant enzyme	Glutathione peroxidase 4
Cytokinin signal transduction	*VvHP*	Histidine-containing phosphotransfer protein	HP
Secondary metabolism	*VvF3H1, VvF3H2*	Isogenes for flavanon 3-hydroxylase	F3H protein
	*VvDMR6*	2-Oxoglutarate and Fe(II)-dependent oxygenase superfamily protein *6* (unknown function)	DMR6-like oxygenase
Pathogenesis-related proteins	*VvGLC*	Hydrolases	β-1,3-Glucanase
	*VvOLP*		Osmotin pathogenesis-related protein 34

**Table 2:** **Genes and their primers and probes used in the gene expression analysis**.

**Gene**	**ENSEMBLE accession N°**	**Primer sequence (5′-3′)**	**Amplicon length (bp)**	**Final concentration (nM)**	**Efficiency**
*VvSUSY4* (Hren et al., 2007)	VIT_11s0016g00470	F: TGTTAAGGCTCCTGGATTTCAATTA	73	900	1.97
		R: AGCCAAATCTTGGCAAGCA		900	
*VvINV2*	VIT_02s0154g00090	F: CCAACCAAGGCGATCTATG	76	900	1.93
		R: TTGAGGCAGTGATGCTGG		900	
		P: AGCTCAGCTCTATGTCTTCAACAATGCTAC		100	
*VvAGPL*	VIT_03s0038g04570	F: GAGTGTAGTTTGGAAGACGATG	111	300	2.03
		R: ACAGGACAGATACCACTTGC		300	
*VvSWEET1*	VIT_18s0001g15330	F: ATACTGTACGCCATCTACTGCAAAA	72	900	1.93
		R: CATGTCCACCGGCTTGTCT		900	
		P: CAGCGGCCAGATTC		250	
*VvSWEET10*	VIT_17s0000g00830	F: TATCTGCGGATTCGGTTCCA	105	300	2.02
		R: ACGCTTAGCGAGAACACGAGAC		300	
*VvSWEET17a*	VIT_05s0077g02260	F: CCTCATATACGCACCGGCAAAA	75	900	2.05
		R: GCTAGAAACCCCACATCCAAGAG		900	
		P: CAGAGCGACGGTTTTG		250	
*VvAPX6*	VIT_04s0008g05490	F: TCGAAGATTTCAAGAATGCCTACATCA	93	900	1.93
		R: CTGTTGAAACGTTGTTGTTCAGGAAT		900	
		P: TTGCACCAGAATTCAC		250	
*VvGPX4*	VIT_07s0104g00320	F: CGTGTAAATGGCCCTGATGCT	68	900	1.96
		R: CCAAGAAATCCACTTTTGTGTGCTT		900	
		P: CCAGTCTACAAATTCC		250	
*VvHP*	VIT_14s0030g00410	F: AGCAACAAATTTTGGCAGC	88	300	1.96
		R: GCCATTTGTCCTCTTGCTC		300	
*VvF3H1* (Gutha et al., 2010)	VIT_04s0023g03370	F: CCAATCATAGCAGACTGTCC	69	300	1.96
		R: TCAGAGGATACACGGTTGCC		300	
*VvF3H2* (Gutha et al., 2010)	VIT_18s0001g14310	F: CTGTGGTGAACTCCGACTGC	129	300	2.05
		R: CAAATGTTATGGGCTCCTCC		300	
*VvDMR6*	VIT_16s0098g00860	F: GCAGGCTCTATGGTTTTTTCC	117	300	2.02
		R: GCTTCATCTTCTCCTCCACC		300	
*VvGLC*	VIT_08s0007g06060	F: CCATCATCAGCTTCCTGGTCAAAA	86	900	1.98
		R: GTCCCGGGTGTTACCAATGTA		900	
		P: CCCCACTGCTTGTTAAC		250	
*VvOLP*	VIT_02s0025g04270	F: TCGCCAGTCTAAACTACTAGG	120	300	1.98
		R: CGTAGAAAAGTTGTTGCATGAG		300	

*F, forward; R, reverse; P, probe*.

Total RNA was isolated using RNeasy Plant Mini kits (Qiagen) and treated with DNase I (Invitrogen), according to Hren et al. (2007, 2009b). RNA was quantified spectrophotometrically using the nanodrop system (Nanodrop Technologies) and reverse transcribed using High Capacity Reverse Transcription kits (Applied Biosystems), as described by Hren et al. (2009b).

In each cDNA sample, the expression of the target genes and the reference genes was determined by qPCR. All of the qPCR reactions were performed on a Viia7 real-time PCR system (Applied Biosystems), in 384-well plate format using universal cycling conditions (2 min at 50°C, 10 min at 95°C, followed by 40 cycles of 15s at 95°C and 1 min at 60°C) with dissociation curve analysis for the SYBR-green-chemistry-based assays (15 s at 95°C, 15 s at 60°C, 15 s at 95°C). QPCR was performed in a final reaction volume of 5 μL, which contained 2 μL cDNA and 3 μL reaction mix. For the SYBR green chemistry, Power SYBR Green PCR Master Mix (Applied Biosystems) was used, and for the TaqMan chemistry, TaqMan Universal PCR Master Mix (Applied Biosystems) was used, with primer and probe concentrations as in Table 2.

The relative expression of the target and reference genes was determined using a standard curve quantification approach. A cDNA pool of all the samples (40 × 5 μL) was used to prepare a standard sample and from it the standard calibration curve. In the Cq/log10 copy number calibration curve arbitrary copy number values were set according to the dilution, because relative and not absolute gene expression was determined. Every sample was tested in two dilutions for every amplicon (gene), and relative copy number was calculated from the calibration curve. The linearity (*R*^2^) and efficiency (*E* = 10[−1/slope]) were determined for each sample. The stability of three reference genes, as *18S, COX* (Hren et al., 2009a) and *ubiquitin* (Gutha et al., 2010), was monitored using NormFinder (Andersen et al., 2004). Based on the data, the geometrical mean of the expression ratios of *18S* and *COX* was used as the normalization factor for the relative transcript target gene-copy number calculations.

Statistically significant differences in relative transcript copy number of each gene between uninfected and infected samples were determined for each time point (*p* < 0.05) using a non-parametric Mann–Whitney *U*-test.

Two-way ANOVA (OriginPro8, OriginLab) was used to evaluate the dependency of gene expression on the two factors of “time of sampling” and “FDp-infection status,” and the interactions between the two factors (*p* < 0.05). The data was log_2_ transformed prior to the analysis. The Tukey test was used for posthoc analysis of ANOVA results.

Principal component analysis (PCA) was also carried out on the log_2_ transformed data, using XLSTAT (Addinsoft).

### Metabolome analysis

Extraction of the samples was performed according to Weckwerth et al. (2004). Briefly, 1 mL methanol/chloroform/water (5:2:1, v/v/v) was added to 80 mg frozen sample powder, vortexed thoroughly, and incubated for 8 min on ice. The sample was then centrifuged at 14,000 × g for 4 min at 4°C, the supernatant was transferred, and 500 μL distilled water was added to it. The mixture was vortexed and centrifuged at 14,000 × *g* for 2 min at 4°C. The methanolic aqueous upper phase was dried under vacuum. Before derivatisation, 5 μL [^13^C]-sorbitol (0.1 g/L) (Sigma-Aldrich) was added to all of the samples as an internal standard, and they were then vacuum dried again. Derivatisation of the samples was carried out in two steps. First, 20 μL 40% (w/v) methoxyamine hydrochloride (Sigma-Aldrich, USA) dissolved in pyridine was added, with an incubation for 90 min at 30°C. After this incubation, 80 μL N-methyl-N-trimethylsilyltrifluoroacetamide silylation mixture (1 ml spiked with 30 μL alkane mixture, of even-numbered C10−C40 alkanes, each at 50 mg/L) was added, and incubated for 30 min at 37°C. The derivatised samples were centrifuged at 14,000 × g for 2 min at room temperature, and 50 μL of supernatant was transferred to gas chromatography vials with micro inserts and closed with crimp caps. Gas chromatography–mass spectrometry analysis was performed according to Mari et al. (2013) on a TRACE Ultra Gas Chromatograph (Thermo Scientific, USA) coupled to a TSQ Quantum GC mass spectrometer (Thermo Scientific, USA). One microlitre of the derivatised samples was injected at a constant temperature of 230°C in split mode (split ratio, 10:1).

The gas chromatography separation was performed on an HP-5MS capillary column (30 m × 0.25 mm × 0.25 μm; Agilent Technologies, Santa Clara, CA, USA) at a constant flow of 1 mL/min helium. The initial oven temperature was set to 70°C and held for 1 min, followed by a ramp to 76°C at 1°C /min, and a second ramp at 6°C/min to 350°C, held for 1 min. The transfer line temperature was set to 340°C, and the post-run temperature to 325°C for 10 min. The mass analyser was used in full-scan mode in the range of *m/z* 40 to *m/z* 600, for a scan time of 250 ms. Electron ionization was used at 70 eV, and the ion source temperature was set to 250°C.

Acquired raw data were processed and validated as follows. Mass spectral deconvolution and retention index (RI) calculation were performed in AMDIS (Stein, 1999) and obtained spectra were matched against an in-house library and a customized version of the Golm Metabolite Database (gmd) (Kopka et al., 2005) including all *m/z* fragments between 40 and 600 Th. Spectral match factors were additionally obtained using the NIST MS Search 2.0 Program algorithm. Confidence levels of identification were given according the Metabolomics Standard Initiative (Sumner et al., 2007), considering compounds as “identified” (MSI Level 1) matched against the in-house library and match factors >750 and RI deviations < 20 (mostly below 5). Compounds matched against an external database (gmd) were considered as “putatively annotated” (MSI level 2) with minimum match factors >750 and RI deviations < 90. Other analytes which could be assigned to compound classes according to specific fragment *m/z* were considered as MSI level 3 (“putatively characterized compound classes”). A peak list and settings are provided in the supplements (Supplementary Table S3). Quantification of obtained compounds was done by extracting peak areas of specific quantification ions (quant *m/z*) in all samples using LC-Quan 2.6.0 (Thermo Scientific, USA). All peaks were additionally inspected manually in means of identification and also peak integration. Response factors were determined as the ratio of the peak area of the compound and the internal standard (quant *m/z*) and were normalized with the mean response factor of the component from all of the samples (normalized response factor).

For statistical analysis, a Shapiro-Wilk test, a non-parametric Mann–Whitney *U*-Test, and PCA (XLSTAT, Addinsoft) were performed.

### Carbohydrate determination

Soluble sugars (glucose, fructose, sucrose), were extracted from 100 mg frozen whole-leaf powder using 500 μL water/ methanol/ chloroform (5:2:1; v/v/v). After 8 min incubation on ice and centrifugation at 14,000 × g for 4 min at 4°C, 250 μL water was added to the transferred supernatant. The mixture was centrifuged at 14,000 × g for 2 min at 4°C. The upper polar phase of the sample was then used for enzyme based spectrophotometric sugar determination using a SUFRG kit (Megazyme International, Ireland). Sucrose, fructose and glucose were determined from 10 to 20 μL of sample, according to the manufacturer's procedure, with half of the reagent amounts used.

The starch in 100 mg frozen leaf powder was determined according to the manufacturer's procedure using a K-TSTA kit (Megazyme International, Ireland).

Each sample was extracted in triplicate for the soluble sugar determination, and in duplicate for the starch determination. Absorbance measurements were determined using a Shimadzu UV-1800 spectrophotometer. The sugar concentrations are given in mg/g fresh weight.

### Enzyme assays

Enzyme assay protocols were based on a newly developed method for simple determination of primary carbohydrate metabolism enzymes from a single extraction with semi-high throughput determination of enzyme activities in microtitre plates (Jammer et al., 2015). Major changes to the original protocols were required, probably due to the high phenolic content of the grapevine extracts that interfered with the enzymatic reactions and detection systems (Covington et al., in review). Details on the modifications and optimizations are listed below.

Three milliliters of extraction buffer (0.5 M MOPS, pH 7.5, 5 mM MgCl_2_, 0.5 mg/mL BSA, 0.05% (v/v) Triton X-100, 25 μM dithiothreitol, 0.1 mM PMSF, 1 mM benzamidine, and 3% (w/v) PEG-4000 (modified from Takayanagi and Yokotsuka, 1997) were added to 0.5 g frozen ground leaf material and allowed to thaw on ice for 10 min, with occasional swirling, followed by centrifugation at 17,000 × g for 10 min at 4°C, to separate the crude extract (supernatant) from the cell-wall fraction.

The AGPase activity was immediately assayed by adding 15 μl fresh supernatant to a reaction mixture containing final concentrations of 100 mM Tris-HCl, pH 8.0, 440 μM EDTA, 5 mM MgCl_2_, 0.1% BSA, 1.5 mM sodium pyrophosphate, 1 mM NADP, 2 mM 3-phosphoglycerate, 1.28 U glucose-6-phosphate dehydrogenase, and 0.432 U phosphoglucomutase, in a final volume of 160 μl at 30°C in a UV-transparent microplate (Corning). Blank reactions were pre-incubated at 30°C for 15 min before addition of 3.125 mM ADP-glucose. The reaction progress was monitored by recording NADPH absorbance at 340 nm in a Synergy MX plate reader at 30°C.

One milliliter of the remaining supernatant was desalted into 20 mM potassium phosphate buffer, pH 7.0, using a PD Miditrap G-25 column (GE Biosciences), according to the manufacturer gravity protocol, followed immediately by the assay for SuSy activity. To reduce interference from background reactions, two separate incomplete reaction mixtures were prepared. Mixture 1 (the “blank”) contained all of the assay components except the leaf extract. Mixture 2 (the “control”) contained all of the assay components except UDP and sucrose. Each mixture was separately pre-incubated at 30°C for 30 min and monitored to verify that NADH absorbance reached a stable plateau. Mixtures 1 and 2 were then combined into a complete assay mixture with final concentrations of 50 mM HEPES/NaOH, pH 7.0, 1 mM EDTA, 2 mM MgCl_2_, 5 mM dithiothreitol, 250 mM sucrose, 1 mM UDP, 1.3 mM ATP, 0.5 mM NAD, 0.32 U glucose-6-phosphate dehydrogenase, 0.672 U hexokinase, 0.56 U phosphoglucose isomerase, and 20 μl desalted grapevine leaf extract, in a final volume of 160 μl. The reaction progress was monitored by recording NADH absorbance at 340 nm, at 30°C. The nINV activity was measured in parallel, with the UDP omitted from the reaction mixture, and was subtracted from the total activity.

The excess desalted extract was stored at −80°C until determination of vacINV activity. For extraction of cwINV, the previously extracted leaf pellet was rinsed with 10 mL ice-cold water and further extracted with 1 ml high-salt extraction buffer (40 mM Tris-HCl, pH 7.6, 3 mM MgCl_2_, 15 mM EDTA, 1 M NaCl) at 4°C, overnight. The material was then centrifuged as above, before desalting on a Miditrap column. The activities of vacINV and cwINV were assayed by adding 10 μl thawed desalted crude extract (for vacuolar invertase) or 5 μl thawed desalted high-salt cell-wall extract (for cell-wall invertase) to 100 mM sucrose, 0.1 M sodium acetate buffer, pH 4.0, in a final volume of 50 μl, and with an incubation for 30 min at 37°C. The reactions were stopped by addition of 200 μl Nelson's alkaline-copper reagent (Nelson, 1944), followed by immersion in a boiling water bath for 20 min. After cooling to room temperature, 200 μl arsenomolybdate reagent (Nelson, 1944) were added, and the mixtures were thoroughly vortexed. The turbid precipitate that formed in all of the samples containing the extract was removed by centrifugation at 17,000 × g for 5 min. Two hundred microlitres of cleared supernatant were loaded into a 96-well plate, and absorbance at 660 nm was recorded. Standard curves were prepared from a 10 mM 1:1 mixture of glucose and fructose. Reducing sugar concentrations were calculated by comparison to a fit of the linear range of the standard curve.

## Results

### SWEET transporters in grapevines

**Figure 1:**
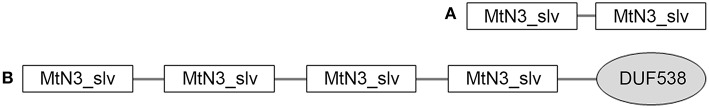
**Structure of the grapevine SWEET proteins. (A)** The common conserved domain architecture of the grapevine SWEET proteins 1-16. **(B)** Architecture of the SWEET17d grapevine protein, with four MtN3/slv domains and a DUF538 domain of unknown function.

To identify grapevine genes that encode SWEET sugar transporters that may be relevant in grapevine yellows diseases, we re-analyzed (Lecourieux et al., 2014) the grapevine genome for their presence (Supplementary Figure S2). This analysis revealed *Vv_SWEET17c* as an additional member of the *MtN3*/*slv*/*SWEET* gene superfamily (Supplementary Table S2). All but one of the SWEET proteins have similar structures, with two MtN3/slv domains placed next to each other. This domain, also known as a PQ loop repeat, comprises a pair of repeats, each of which spans two transmembrane helices connected by a loop (https://www.ebi.ac.uk/interpro/entry/IPR006603). SWEET17c has four MtN3/slv domains and a DUF538 domain of unknown function at the end of the sequence (Figure 1). The grapevine *VvSWEET* paralogs with 27 to 80% identity classify into four clades (Supplementary Figure S2), as previously shown for other plant genomes (Chen et al., 2010; Lecourieux et al., 2014). Based on literature data about the known or putative functions and their possible involvement in grapevine yellows diseases of the SWEETs, genes *VvSWEET1, VvSWEET10*, and *VvSWEET17a* were selected for expression analyses in the present study. In particular, their respective homologs in *Arabidopsis* encode a glucose transporter that can mediate both uptake across the plasma membrane and efflux into the endoplasmic reticulum, and that can function as a bidirectional uniporter/facilitator that is largely dependent on pH (i.e., *VvSWEET1*), a putative sucrose transporter (i.e., *VvSWEET10*) and a putative tonoplast fructose transporter (i.e., *VvSWEET17a*).

### Expression of the selected genes upon infection with FDp

Previous studies on grapevines infected with BNp have shown that genes encoding proteins from various metabolic pathways have statistically significant differential expression due to phytoplasma infection (Hren et al., 2009a; Landi and Romanazzi, 2011). To obtain better insight into these metabolic pathways in grapevines infected with FDp, we analyzed the seasonal temporal dynamics of transcription levels of these genes. Given the association of phytoplasma infections with carbohydrate metabolism, we also included in this study selected genes that encode sugar transporters from the SWEET family that have not before been examined in grapevines. To address the time course of regulation during infection, we examined the expression of the chosen genes in leafvein-enriched tissues every month from May to August.

**Figure 2:**
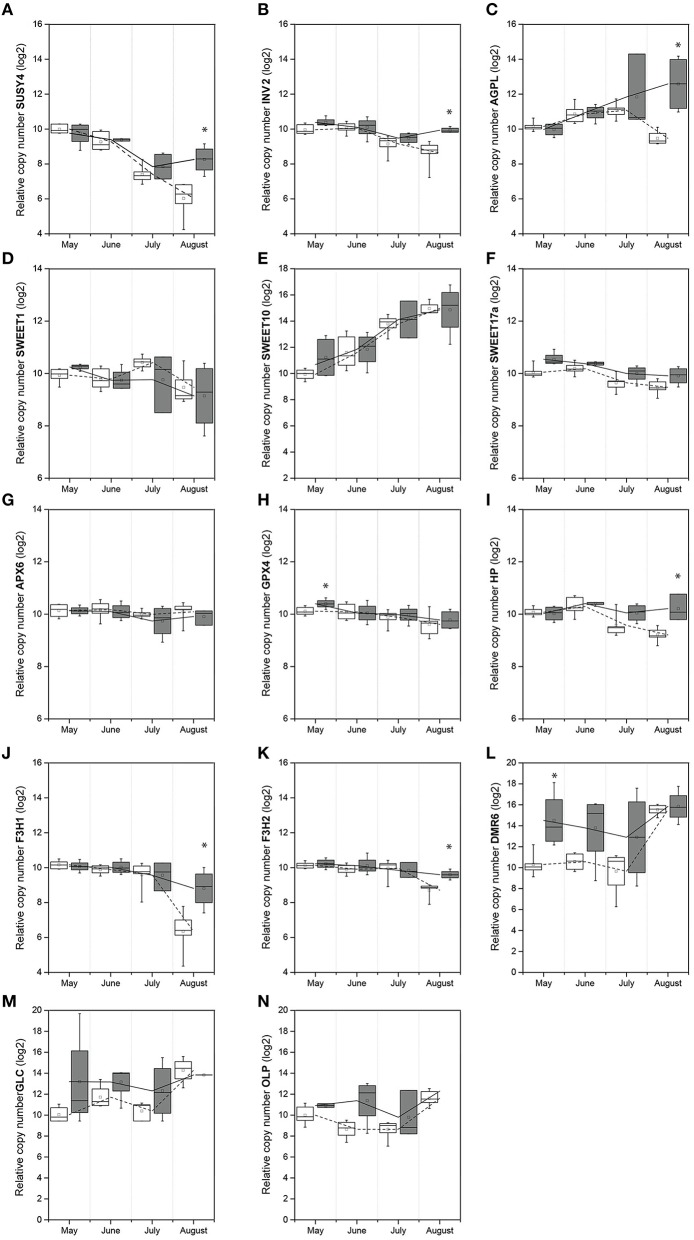
**Temporal expression of genes in leaf vein-enriched samples from uninfected and FDp-infected grapevines: (A) *VvSUSY4*; (B) *VvINV2*; (C) *VvAGPL*; (D) *VvSWEET1*; (E) *VvSWEET10*; (F) *VvSWEET17a*; (G) *VvAPX6*; (H) *VvGPX4*; (I) *VvHP*; (J) *VvF3H1*; (K) *VvF3H2*; (L) *VvDMR6*; (M) *VvGLC*; (N) *VvOLP***. The values are log_2_ transformed and normalized using a geometric mean of 18S and COX Cq values. ^*^Significantly different relative expression levels of uninfected and FDp-infected samples (*p* < 0.05; Mann–Whitney *U*-tests); white box plots, uninfected; gray box plots, infected; line, median; square, mean; box, 25th and 75th percentiles; whiskers, minimum and maximum; lines (dashed line, uninfected; solid line, infected) represent the mean relative expression.

The overall trend of *VvSUSY4* and *VvINV2* gene expression in uninfected samples decreased throughout the season (Figures 2A,B). However, the transcript levels in infected plants only decreased until July, but then increased in August. Also, in August, the *VvSUSY4* and *VvINV2* transcript levels in infected samples were significantly higher than in uninfected ones. *VvAGPL* (Figure 2C) was up-regulated from May to July, and its expression continued increasing in the infected samples in August, while in the same period *VvAGPL* transcript levels dropped steeply in uninfected leaf vein-enriched samples. Two-way ANOVA was used to test whether the gene expression depended on two factors—infection with FDp and the time point in the growing season—and to test whether there were any interactions between these two factors (Table 3). This analysis revealed that the expression of *VvSUSY4, VvINV2*, and *VvAGPL* was associated with both time and infection (Table 3).

**Table 3:** **Two-way ANOVA analysis for effects of time of sampling (from May to August) and FDp infection of grapevines on leaf-vein expression of the selected genes, and the interactions between these factors**.

**Gene**	**ANOVA** ***p***		
	**Time**	**FDp infection**	**Interaction Time × FDp infection**
*VvSUSY4*	1.8E-10^***^	0.006^**^	0.001^**^
*VvINV2*	4.8E-05^***^	0.001^**^	0.029^*^
*VvAGPL*	0.014^*^	0.003^**^	0.002^**^
*VvSWEET1*	0.034^*^	0.390	0.398
*VvSWEET110*	9.5E-08^***^	0.482	0.915
*VvSWEET17a*	5.3E-06^***^	1.0E−4^***^	0.606
*VvAPX6*	0.290	0.264	0.870
*VvGPX4*	0.004^**^	0.213	0.713
*VvHP*	0.001^**^	0.001^**^	0.012^*^
*VvF3H1*	1.7E-08^***^	0.014^*^	0.001^**^
*VvF3H2*	1.8E-05^***^	0.049^*^	0.058
*VvDMR6*	0.002^**^	0.002^**^	0.308
*VvGLC*	0.280	0.106	0.638
*VvOLP*	2.0E-04^***^	0.002^**^	0.232

* p < 0.05;

** p < 0.01;

*** *p < 0.001*.

The overall expression patterns of the *VvSWEET* genes (Figures 2D–F) were similar in the uninfected and infected samples. However, their expression significantly changed over time (Table 3), and the *VvSWEET17a* transcript was significantly more abundant in the infected samples, although the fold change was small (Table 3).

The expression patterns for genes *VvAPX6* and *VvGPX4* encoding antioxidant enzymes were also similar in uninfected and infected leaf vein-enriched samples, although *VvGPX4* was up-regulated at the beginning of the growing season in infected samples (Figures 2G,H; Table 3). *VvHP* was significantly up-regulated in infected samples in August, and its expression was significantly affected by both time and FDp infection (Figure 2I; Table 3). The expression of *VvF3H1* and *VvF3H2* decreased in all of the leaf vein-enriched samples during the growing season, but was significantly higher in the infected samples in August (Figures 2J,K; Table 3). However, the interaction between the time and infection factors only reached significance for *VvF3H1* (Table 3). The expression of *VvDMR6* was higher in infected vein-enriched samples compared with uninfected ones, especially in May (Figure 2L). By August, *VvDMR6* expression had increased in uninfected samples, to a level such that the difference was no longer observed. The expression pattern of *VvGLC* was similar in uninfected and infected leaf vein-enriched samples (Figure 2M, Table 3). Tracking of *VvOLP* expression throughout the season revealed higher transcript abundance from May to July compared to uninfected samples, with a decrease in July and subsequent increase in August (Figure 2N, Table 3).

Taken together, both the season and infection with FDp affected the expression of genes involved in carbohydrate metabolism (*VvSUSY4, VvINV2, VvAGPL*) in leaf vein-enriched tissues. The same was true for genes *VvF3H1* and *VvF3H2* that encode flavanone-3-hydroxylase, a key enzyme involved in the biosynthesis of flavonoid compounds, and the gene *VvHP* from the cytokinin signaling pathway. It is of note that most of the analyzed genes were up-regulated in the infected samples in late summer, and thus it is likely that they are associated with development of symptoms. However, *VvGPX4* and *VvDMR6* showed differential expression in spring, before symptom development, as they were both significantly up-regulated in May.

**Figure 3:**
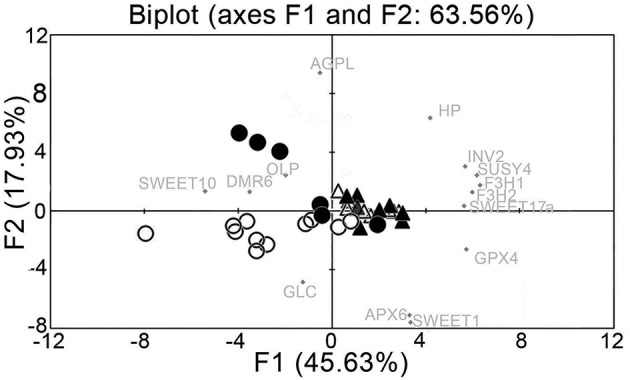
**Principal component analysis of gene expression data from uninfected and FDp-infected leaf vein-enriched samples**. Open triangle, spring uninfected; solid triangle, spring infected; open circle, summer uninfected; solid circle, summer infected.

Principal component analysis for all of the data on gene expression from uninfected and infected samples partially resolved the sanitary status of the samples and their spring or summer origin (Figure 3). The spring and summer samples were separated by the first principal component (PC1), which contributed 45.63% of the total variation. Summer infected and uninfected samples were separated by the second principal component (PC2), which explained 17.93% of the total variation. The data for the analysis also included two infected plants that lost their disease symptoms from spring to summer, and in which FDp was no longer detectable in the following growing season (Supplementary Figure S1). The gene expression results from these recovered grapevines likely contribute to the poorer discrimination. Comparison of the expression of these 14 genes shows that the main contributors to the first component were *VvF3H1, VvSUSY4, VvF3H2, VvGPX4, VvINV2*, and *VvSWEET17a* (Figure 3), which correlates with the ANOVA results that showed their dependence on time (Table 3). On the other hand, the main contributors to the second component were *VvAGPL, VvSWEET1, VvAPX6, VvHP*, and *VvGLC* (Figure 3).

### Leaf vein-enriched tissue-specific metabolic response of grapevine to FDp

Metabolome analysis of the leaf vein-enriched tissues that were sampled in summer allowed the detection of 247 compounds. A total of 78 compounds were annotated or putatively annotated, and 15 of these differed significantly (*p* < 0.05) between infected and uninfected samples by Mann–Whitney *U*-test (Table 4, Supplementary Table S3). The majority of the compounds increased in infected samples.

**Table 4:** **The annotated or putatively (P) annotated compounds in uninfected summer leaf-vein samples of cv. “Modra frankinja” compared to those from FDp-infected grapevines**.

**Compound group**	**Compound**	**FDp/uninfected ratio**
Amino acids	Valine	1.51^↑^
	Leucine	1.72
	Proline	3.17
	Glycine	1.01
	Alanine	1.57
	β-Alanine	2.27
	Serine	1.50
	Threonine	1.56^↑^
	Aspartate	1.76
	Pyroglutamate	1.91^↑^
	Glutamate	1.09
	Phenylalanine	2.19^↑^
	Glutamine	0.45
Acids, esters, lactones	Pentahydroxyhexanoic acid 1	0.94
	Pentahydroxyhexanoic acid 2	0.79
	Pentahydroxyhexanoic acid 3	0.60^↓^
	Pentahydroxyhexanoic acid 4	0.68
	Pentahydroxyhexanoic acid-1,4-lactone (P)	0.76^↓^
	Maleate	1.23
	Fumarate	0.80
	Malate	1.41
	Citramalate (P)	1.64^↑^
	2-Oxoglutarate	2.41^↑^
	Citrate	1.20
	Isocitrate	1.15
	Succinate	1.48^↑^
	Ascorbate	2.20
	Dehydroascorbate	1.10
	Pyruvate	4.06^↑^
	Palmitic acid	0.43
	Stearic acid	0.42
	Glycerate	0.90
	Glycolate	0.95
	Threonic acid-1,4-lactone (P)	0.79
	Threonate	1.00
	Phosphoric acid	1.71
	2,4-Dihydroxy-butanoic acid (P)	1.61
	Malonic acid	1.75^↑^
	Erythronic acid	1.020
	Ribonic acid	1.92
	Lyxonic acid	1.20
	Arabinonic acid (P)	1.10
Sugars, sugar derivatives	Aldopentose 1	1.16
	Aldopentose 2	1.34
	Aldopentose 3	1.45
	Ketopentose	1.12
	Pentose alcohol1	1.21
	Pentose alcohol 2	0.94
	Fructose	1.23
	Galactose or Mannose	1.45
	Glucose	1.19
	Fructose-6-P (P)	1.75^↑^
	Hexose-6-P	1.75^↑^
	Hexose-6-P	1.41
	Sucrose	0.80
	Saccharide 1	0.71
	Saccharide 2	0.76
	Saccharide 3	0.40
	Saccharide 4	0.58
	Saccharide 5	0.85
	Saccharide 6	0.33
	Saccharide 7	1.63
	Saccharide 8	1.38
	Saccharide 9	1.07
	Saccharide 10	3.61
	Saccharide 11	2.018
	Inositol	0.89
Phenols, alcohols, ketones	Epicatechin (P)	2.09
	Catechin (P)	1.65
	Flavonoid	0.74
	Caffeate (P)	1.28
	Salicylic acid-glucopyranoside (P)	7.53
	Salicylate (P)	13.13^↑^
	Quinate	0.89
	Shikimate	0.689
	2-Methyl-1,3-butanediol (P)	1.75
	Glycerol	1.73^↑^
Nitrogen compounds	Ethanolamine	1.97

*The infected to uninfected ratios represent the average normalized response factors of each compound between the infected and uninfected samples*.

↓ indicates amounts that were significantly lower in infected samples than in uninfected ones (p < 0.05);

↑ *, similarly, but significantly higher (p < 0.05)*.

Significant increases in fructose-6-phosphate, hexose-6-phosphates and several metabolites of the tricarboxylic acid (TCA) cycle are noteworthy. The levels of valine and threonine in the leaf vein-enriched samples were significantly higher in grapevines infected with FDp and those of serine, leucine, phenylalanine and β-alanine were also increased. In addition, a 7.5-fold increase in salicylic acid-glucopyranoside and 13-fold significant increase in salicylate (Table 4, Figure 6B, Supplementary Table S3) were also associated with infected samples. In contrast, the peak corresponding to pentahydroxyhexanoic acid or its derivates was higher in uninfected grapevine samples than in samples infected with FDp.

**Figure 4:**
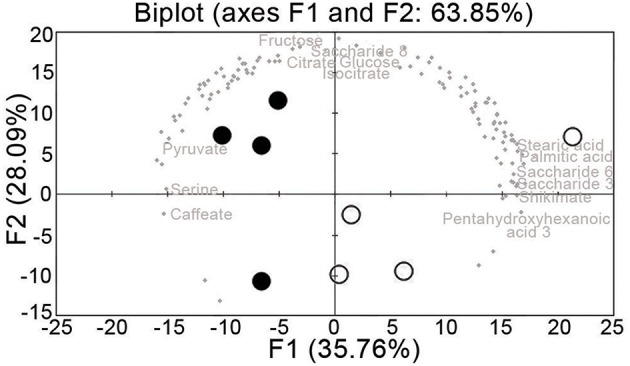
**Principal component analysis of the leaf vein-enriched metabolome data from uninfected and FDp-infected grapevine samples**. Open circle, summer uninfected; solid circle, summer infected.

Principal component analysis was performed on the metabolomic dataset. The sanitary status can be resolved by PC1, which contributed 37% of the total variation (Figure 4). Among the annotated metabolites, the main contributors to PC1 were: palmitic acid, pentahydroxyhexanoic acid, shikimate, stearic acid, saccharides 3 and 6, caffeate, serine, and pyruvate (Figure 4). The main contributors to PC2 were fructose, glucose, saccharide 8, citrate, and isocitrate.

### Carbohydrate content

**Figure 5:**
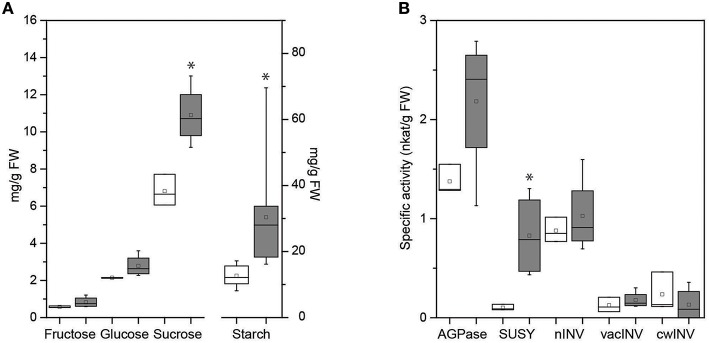
**Box plot of the carbohydrate concentrations (A) and the specific activities of ADP-glucose pyrophosphorylase (AGPase), sucrose synthase (SuSy), and neutral (nINV), vacuolar (vacINV), and cell-wall (cwINV) invertases (B) in summer whole-leaf samples of uninfected (white boxes) and FDp-infected (gray boxes) grapevines**. Line across the box, median; square, mean; box, 25th and 75th percentiles; whiskers, minimum and maximum values. ^*^p < 0.05 (Student's *t*-tests).

As the main transport carbohydrate in plant phloem, the major compound in all vein-enriched samples was sucrose. Its amount was in great excess over other compounds and led to detector saturation in GC-MS. Thus, we evaluated the concentrations of the key soluble carbohydrates and starch by a standard biochemical approach in whole-leaf samples, which also include the leaf mesophyll. Similar to the data for vein-enriched tissues, there was a trend toward higher concentrations of fructose and glucose in infected whole leaves (Figure 5). However, the amount of starch, which was not detected in the vein-enriched samples, was significantly higher in infected whole leaves than in uninfected ones (Figure 5), which confirms its accumulation in the mesophyll during FDp infection. In addition, the significantly higher concentration of sucrose in infected whole-leaf samples (Figure 5) compared to similar concentrations in uninfected and infected leaf vein-enriched samples (Table 4) also indicates its mesophyll accumulation.

### Enzyme activities

Analysis of the impact of FDp on metabolite levels in whole leaves was complemented by analysis of key enzymes involved in primary carbohydrate metabolism. Three enzymes in particular, vacuolar invertase (vacINV), sucrose synthase (SuSy), and ADP-glucose pyrophosphorylase (AGPase) are of particular interest because their transcript levels are increased in BNp-infected grapevine leaves (Hren et al., 2009a). Jammer et al. (2015) have developed a new simple and robust method to determine the signature of the key enzymes in primary carbohydrate metabolism from a single extraction, followed by semi-high-throughput determination of 13 different enzyme activities in microtitre plates. This technique offers a number of significant advantages for plant research, including the ability to assay multiple enzymes from only one extract when limited sample is available. The protocol was successfully tested with a number of species; however, biochemistry in grapevine is notoriously difficult due to high levels of interfering compounds, including phenolics. As a result, a number of alterations to the original protocol were required in adapting high-throughput techniques to grapevine (Covington et al., in review).

The activities of the enzymes AGPase and SuSy, and the nINV, vacINV and cwINV isoenymes as key determinants of the levels of the main soluble sugars (glucose, fructose, sucrose) and starch were measured in the summer whole-leaf samples previously used to measure carbohydrates (Figure 5B). The activities of both SuSy and AGPase were higher in infected whole-leaf samples than in uninfected ones, although due to the limited sample size, only the difference in SuSy activity was statistically significant. SuSy was induced eight-fold from 0.10 nkat/g FW to 0.83 nkat/g FW in FDp-infected leaves. In contrast, activities of the three invertase isoenzymes (i.e., nINV, vacINV, cwINV) were unaffected by FDp infection at the time of measurement. A similar trend was observed in whole-leaf samples from the following summer, although the small number of infected samples that year precluded meaningful statistical analysis (Supplementary Table S4).

## Discussion

In this study, we have addressed dynamics of host responses to phytoplasma infections under natural vineyard conditions in leaf vein-enriched samples and in whole grapevine leaves with non-targeted determination of metabolic fingerprints that was complemented by determination of major carbohydrates and key enzyme activities and mRNA levels of representative genes involved in primary and secondary metabolism and defense responses. While the veins also contain phloem tissues in which phytoplasma thrive (Christensen et al., 2004) and are thus expected to be where the first metabolic changes take place after infection, the whole source leaf includes the photosynthetic parenchyma cells in the mesophyll tissue and is believed to be the site of carbohydrate accumulation when plants are infected with phytoplasma (Lepka et al., 1999; Maust et al., 2003; Junqueira et al., 2004; Musetti et al., 2013). Although for technical reasons the separation of tissues was not complete in our experiments, genes followed in our study that are involved in sucrose transport and metabolism, as well as genes encoding pathogenesis-related-proteins (PRP) from class 5 have been shown to be expressed both in BNp-infected grapevine vein-enriched and phloem tissues; only the sensitivity of detection was higher in phloem (Santi et al., 2013b). In addition, comparison of the metabolic profiles of leaf and phloem sap from mulberry infected with phytoplasma found that the infection had a greater effect on the metabolome of the phloem sap than on that of the whole leaf (Gai et al., 2014).

### Pre-symptomatic grapevine responses to FDp infection

We have shown that the FDp titre is extremely low at the beginning of the growing season, and that it increases in close association with the expression of symptoms afterwards (Prezelj et al., 2013). Therefore, in general, detection of FDp is either impossible or is unreliable in asymptomatic plants. To prevent the spread of FDp, it would be beneficial to have a plant-host marker for accurately sensing FDp even when its titre is below the limit of detection. Our analysis here detected only the genes *VvGPX6* and *VvDMR6* differentially expressed in infected plants in spring before the development of symptoms. While the enzyme *VvGPX6* is a more general component of host plant defense responses related to oxidative stress (Foyer and Noctor, 2005), the biological role of *VvDMR6*, which encodes a 2-oxoglutarate and Fe(II)-dependent oxygenase, is uncertain at the moment. However, it has been shown that in *Arabidopsis* lacking a functional *DMR6*, susceptibility to downy mildew is reduced (Van Damme et al., 2008) and has been suggested that DMR6 acts as a suppressor of plant immunity (Zeilmaker et al., 2015). Similar early seasonal up-regulation of *VvDMR6* has also been observed during BNp-infection, as well as in grapevines recovered from bois noir (Dermastia et al., 2015). Although it remains to be determined how specific is the early spring expression of *VvDMR6* in terms of phytoplasma diseases, it may be added to the list of potential early markers of grapevine yellows.

### Modified primary grapevine metabolic pathways during infection with FDp

**Figure 6:**
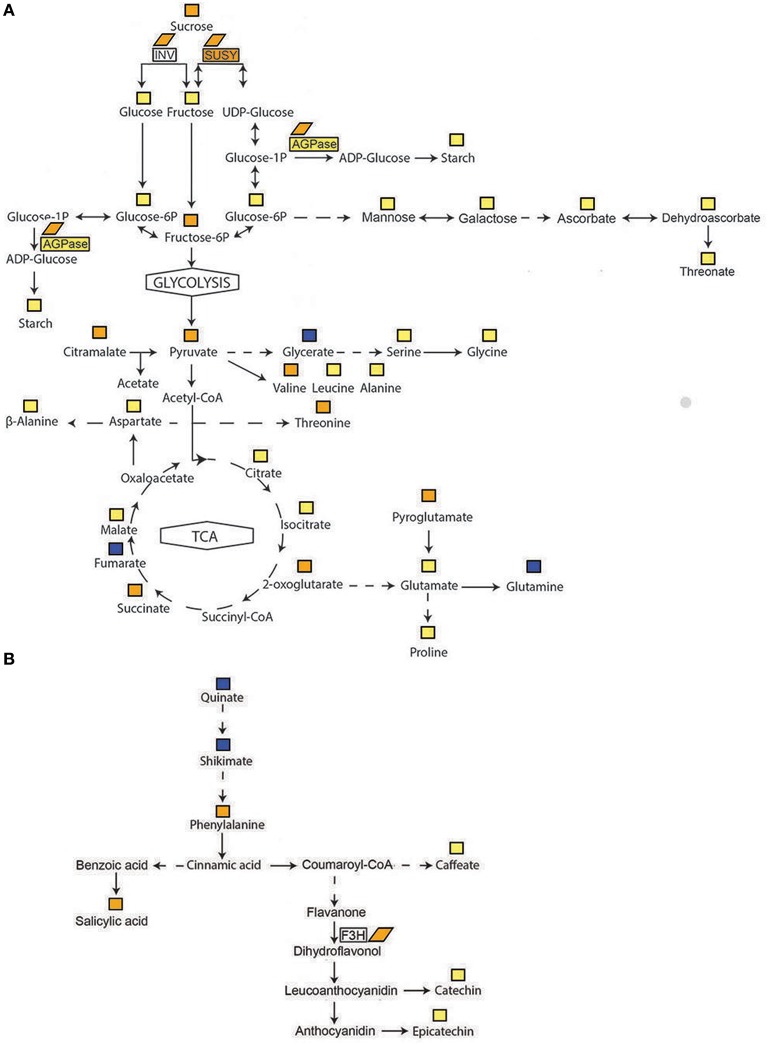
**Schematic representation of the metabolites in the primary (A) and in phenylpropanoid metabolism (B) pathways affected by FDp infection of grapevine cv**. **“Modra frankinja” together with some enzymes involved in their conversions and followed in this study**. Blue square, metabolite in uninfected samples > than metabolite in infected samples; yellow square, metabolite in infected samples > than metabolite in uninfected samples; orange square, metabolite in infected samples > than metabolite in uninfected samples at *p* < 0.05; open rectangle; enzyme activity in infected samples not significantly different than in uninfected samples; yellow rectangle, enzyme activity in infected samples > than in uninfected samples; orange rectangle, enzyme activity in infected samples > than in uninfected samples at *p* < 0.05; orange rhomb, expression of gene encoding enzyme in infected samples > than in uninfected samples at *p* < 0.05.

It is assumed that modifications of the primary metabolism of the host plant during infection with pathogen are associated with cellular requirements for plant defense responses (Rojas et al., 2014). Previously, down-regulation of photosynthesis has been demonstrated as a common response in several phytoplasma-plant interactions (Leon et al., 1996; Bertamini and Nedunchezhian, 2001; Hren et al., 2009a), including grapevine infected with FDp (Margaria and Palmano, 2011; Margaria et al., 2013; Vitali et al., 2013). Our observation of an increase in fructose-6-phosphate, pyruvate and metabolites of the TCA cycle in vein-enriched grapevine tissue implies induction of the energy associated network (Table 1, Figure 6A, Supplementary Table S3). It has been shown in other plant pathogen infections that the increase in respiratory metabolism, as well as activities of carbohydrate transporters and sucrolytic enzymes, is coupled with promotion of the favorable energy balance for plant defense (Rojas et al., 2014). The general and energy-demanding upper part of glycolysis is encoded in all known phytoplasma genomes (Kube et al., 2012), including the FDp genome (Carle et al., 2011). However, phytoplasma lack hexokinase and the sugar-specific phosphotransferase system that mediates entry of phosphorylated hexose into glycolysis. This problem would be overcome by an uptake system that allowed utilization of phosphorylated hexoses from the phytoplasma host. It has been suggested that phytoplasma can use sucrose and trehalose compounds from phloem sap, using the phosphoglucose isomerase encoded in all known phytoplasma genomes. However, this step would not be necessary if fructose-6-phosphate is available (Kube et al., 2012). In association with this suggestion, fructose-6-phosphate might be also utilized by FDp. It could enter the FDp glycolysis pathway if it were converted to fructose-1,6-bisphosphate by plant phosphofructokinase. We have previously shown that the gene encoding this enzyme is up-regulated in BNp-infected grapevine (Hren et al., 2009a).

### VvSWEET17a changed during the FDp-infection

Little information is available about sugar transporters involved in phytoplasma pathogenicity. We examined three sugar transporters from the SWEET class (Chen et al., 2010, 2012; Chen, 2014; Eom et al., 2015) at the transcriptional level for possible association with the development of flavescence dorée. Specifically, in uninfected and infected grapevine leaf vein-enriched tissues we followed gene expression of *VvSWEET1* from clade I, which is involved mainly in transport of monosaccharides, *VvSWEET10* from clade III, which is involved mainly in sucrose transport (Chen et al., 2010, 2012), and *VvSWEET17a* from clade IV, which is involved in vacuolar transport of fructose (Guo et al., 2014). Irrespective of infection, the transcript levels of all three of these *SWEET* genes showed significant changes throughout the growing season, although only the gene expression of *VvSWEET17a* was affected by FDp infection, being higher in the infected grapevines. Expression patterns of clade III-*SWEET* orthologs in *Arabidopsis* and rice show induction by biotrophic bacteria and by fungi (Chen, 2014), which suggest a certain level of pathogen dependence on the SWEETs. However, prior to the present study, there were no reports of pathogen association with SWEETs from clade IV. Further studies are now needed to confirm a role for *VvSWEET17a* in phytoplasma infection. Another sucrose transporter involved in grapevine interaction with phytoplasmas is *SUC27*, which is down-regulated in phloem upon infection with BNp, indicating reduced phloem loading (Santi et al., 2013b).

### Role of sucrose synthase in FDp-grapevine interaction

The accumulation of sucrose and starch in grapevine leaves infected with FDp corroborated similar findings in other plants infected with phytoplasmas (Lepka et al., 1999; Maust et al., 2003; Gai et al., 2014). Carbohydrate accumulation is presumably related to physically obstructed phloem loading and transport due to callose depositions in sieve tubes, as has been shown in *V. faba* infected with FDp (Musetti et al., 2013). The formation of callose plugs resembles the responses of tobacco leaves during an incompatible interaction with *Phytophtora nicotiane* (Scharte et al., 2005). A supply of UDP-glucose for rapid biosynthesis of callose plugs in the sieve pores is provided by the enzyme sucrose synthase (SuSy), which catalyzes sucrose breakdown to UDP-glucose and fructose *in planta* and is localized in both companion cells and sieve elements of the phloem (Koch, 2004). The specific role of sucrose synthase in FDp-mediated responses is supported by an 800% increase in enzymatic activity of SuSy, reported here for the first time in FDp-infected grapevines. The increase in activity upon infection was 3.5-fold higher than that reported for SuSy-overexpressing potato tuber (Baroja-Fernández et al., 2009). This very high SuSy activity suggests possible other important roles for SuSy in FDp-grapevine interaction that are not necessarily mutually exclusive with providing precursors for callose plug formation.

Impaired phloem loading and transport, together with carbohydrate accumulation and metabolic feedback inhibition of photosynthesis in source organs, is indicative of a source-to-sink transition that modifies the mechanism of sugar transport and partitioning at the whole plant level (Lemoine et al., 2013). Slight but significant inhibition of photosynthesis in source leaves has been demonstrated in grapevine infected with BNp from July to September (Endeshaw et al., 2012). The source-to-sink switch is regulated by cytokinins (Roitsch and Ehness, 2000). At the moment it is not known whether a significant transcript increase of the gene *VvHP* from the cytokinin signaling pathway is related to this transition.

Several plant pathogens are able to manipulate host metabolism to turn infected tissues into a carbohydrate sink that provides them with hexoses (Berger et al., 2007). Such a transition is usually characterized by increased activity of invertases, which irreversibly hydrolyze sucrose to glucose and fructose (Roitsch and González, 2004). Three groups of invertases can be distinguished: neutral (nINV), acid insoluble bound to the cell wall (cwINV), and acid soluble localized in the vacuole (vacINV). In leaf samples infected with FDp, the activities of the different invertase isozymes were not significantly higher than in healthy controls. In accordance with these results healthy and infected samples had similar amounts of the breakdown products of the invertase reaction, glucose and fructose. On the other hand, the expression of the *VvINV2* gene that encodes a vacuolar invertase significantly changed throughout the growing season in both healthy and FDp-infected vein-enriched samples, and was up-regulated in these FDp-infected samples in August. Post-translational regulation of the corresponding invertase activity by a proteinaceous inhibitor might account for this discrepancy, and transcript and protein abundances are indeed often poorly correlated (Stitt and Gibon, 2014). A slight increase in transcription of the gene encoding cwINV has been recorded in grapevine infected with BNp by Santi et al. (2013a), but not in a microarray study of the same interaction (Hren et al., 2009a).

In contrast with invertases, significantly higher abundance of the *VvSUSY4* transcript in infected vein-enriched tissues in August agreed with the substantial increase in activity of SuSy at the same time in the growing season. Reverse correlation between the expression of SuSy and acid invertase has been confirmed in SuSy-overexpressing potato tubers (Baroja-Fernández et al., 2009). The findings of our study underline the significance of SuSy in phytoplasma-plant interaction, as has been recently suggested for maize infected with maize bushy stunt phytoplasma (Brzin et al., 2011). In phloem conditions of high sucrose and low fructose, as seen from the metabolome analysis, SuSy likely operates as a sucrose-degrading enzyme. However, continous mobilization of sucrose via SuSy depends upon removal of fructose (Geigenberger et al., 1993). Fructose might be utilized directly by FDp as has been proposed for *Spiroplasma citri*, another member of the *Mollicutes* class (Gaurivaud et al., 2000). Fructose utilization such as that proposed for spiroplasmas could impair sucrose loading into sieve tubes by companion cells, resulting in accumulation of carbohydrates in source leaves, as is seen in FDp-infected grapevines. However, a model of *S. citri* operation involved a putative role for acid invertase, although this enzyme was not studied in the system (André et al., 2005). Further studies are needed to ascertain the role of fructose in FDp pathogenicity, as well as a putative role for the vacuolar fructose transporter *VvSWEET17a*, which was up-regulated during FDp infection and has been suggested to function predominantly in sink organs (Chardon et al., 2013).

### Induced starch accumulation in infected leaves

Expression of the *VvAGPL* gene was significantly higher in the leaf vein-enriched tissues of infected grapevines than in uninfected samples, and thus *VvAGPL* was an important contributor to the uninfected/infected cluster formation in the PCA. *VvAGPL* encodes the large, regulatory subunit of ADP-glucose-pyrophosphorylase (AGPase), which is a rate-limiting enzyme in starch biosynthesis (Ballicora et al., 2004). In agreement with the abundant *VvAGPL* transcript in infected leaves, there was also a trend toward higher AGPase activity and significantly higher starch concentration. Interestingly, in grapevines infected with *Plasmopora viticola*, the expression of *VvAGPL* was lower in comparison with uninfected plants, while the AGPase activity was higher (Gamm et al., 2011), consistent with the multiple levels of post-translational control known for AGPase. High starch concentration in phytoplasma-infected mulberry leaves has been explained by lower expression of genes and/or lower activity of enzymes for the degradation of starch (Gai et al., 2014). In contrast with this observation we have reported up-regulation of the gene encoding α-amylase in grapevines infected with BNp (Hren et al., 2009a). Although in our experimental system we were not able to follow the transient starch degradation, the possibility of its phosphorolityc degradation leading to the increase in hexose-6-phosphates observed here, could not be excluded. However, the expression of genes encoding the proposed enzymes involved in this process, glucan, water dikinase and β-amylase (Smith et al., 2005) were not differentially expressed in grapevines infected with BNp (Hren et al., 2009a).

### Modified secondary grapevine metabolic pathways during infection with FDp

The source-sink transition upon pathogen infection is typically linked to coordinated defense responses and enhances the expression of defense-related genes and production of secondary metabolites (Ehness et al., 1997; Roitsch, 1999; Rojas et al., 2014). Our study is in agreement with these suggestions. Of interest was the expression of *VvGLC* encoding a PRP-2 β-1,3-glucanase and *VvOLP* encoding a member of the PRP-5 class. Both genes were slightly induced in spring, and throughout the season their expression remained higher compared to uninfected samples. In contrast in late summer the amount of *VvOLP* protein product was 77-times induced in the FDp-infected grapevine cv. “Nebbiolo,” which is known to be less susceptible to FDp than are other cultivars (Margaria and Palmano, 2011). In addition, *VvGLC* and members of the *PRP5* class are up-regulated in symptomatic FDp grapevine of cv. “Barbera” (Gambino et al., 2013) as well as in BNp-infected grapevines (Hren et al., 2009a; Landi and Romanazzi, 2011; Santi et al., 2013b). These *PR-2* and *PR-5* genes are commonly used as molecular markers for salicylic-acid-dependent systemic acquired resistance signaling, and their expression is co-ordinately regulated by salicylic acid (Frías et al., 2013). It has been suggested that BNp induces salicylic-acid-dependent systemic acquired resistance in leaves of infected tomatoes and grapevines (Ahmad et al., 2015; Dermastia et al., 2015). The up-regulation of *VvPR-2* and *VvPR-5* genes, together with increased levels of salicylic acid and salicylic-acid-glucopyranoside (7.52-fold and 13.13-fold, respectively) seen here, support this idea in these infected grapevines. The less-pronounced response to infection with FDp of cv. “Modra frankinja” compared to cv. “Nebbiolo” and grapevines infected with BNp points to the more aggressive nature of FDp and stronger susceptibility of the examined cultivar. Although the FDp-infection triggers a defense response, this response is not enough to resist the infection.

In addition, two genes, *VvF3H1* and *VvF3H2*, involved in biosynthesis of flavonoids and induced by sucrose (Solfanelli et al., 2006), were also up-regulated in infected vein-enriched tissues. Flavonoids have several roles, which include responses of grapevines to infections with FDp and BNp (Hren et al., 2009a; Landi and Romanazzi, 2011; Rusjan et al., 2012a,b; Margaria et al., 2014). It is of note that the number of transcripts of *VvF3H1* and *VvF3H2* in cv. “Modra frankinja” decreased throughout the season in both uninfected and infected samples, but remained significantly higher in the infected ones in August, compared to the uninfected samples. On the other hand, levels of the *VvF3H2* transcript steadily increased from spring to August although they were consistently low in uninfected cv. “Barbera” leaves compared to leaves infected with FDp from subgroup C (Margaria et al., 2014). In cv. “Nebbiolo,” *VvF3H2* expression is less pronounced, and the transcript abundance is at its highest in mid-July (Margaria et al., 2014). It appears therefore that the observed differences might be both phytoplasma strain- and cultivar-related. Our metabolome analysis additionally showed two phenolics downstream of the proanthocyanidin pathway to be increased in infected samples: epicatechin and catechin (Figure 6B). However, the GC-MS based metabolite profiling approach used in this study is usually more amenable for detection of primary metabolites (i.e., small molecules). Hence, future studies should also include LC-MS analyses to get an even more comprehensive picture of changes in secondary metabolism. In agreement with our findings of the impact of FDp infection on secondary metabolite profiles is the generally observed co-ordination of source-sink transition and defense responses after pathogen infections. Among these defense responses is regulation of secondary metabolism, including the shikimic acid (Rolland et al., 2002) and phenylpropanoid (Ehness et al., 1997) pathways.

## Concluding remarks

In this study, we have demonstrated that infection of grapevines with FDp significantly affects the expression of key genes of primary and secondary metabolism and the metabolome of leaf vein-enriched tissues (Figure 6). Under consideration of the known impacts of phytoplasma infection, such as callose deposition and repression of photosynthesis, our data support the model that FDp infection initially results in a block of sugar export through effects on phloem transport and metabolism. The accumulation of soluble carbohydrates results in feedback inhibition of photosynthesis that causes a source-sink transition, in agreement with the general assumption that the metabolic situation of photoautotrophic source leaves is not very well-suited for defense (Roitsch, 1999; Rojas et al., 2014). In parallel to the responses of primary carbohydrate metabolism genes, sink-specific secondary metabolism pathways involving genes and metabolites of the shikimic acid and oxidative pentose phosphate pathway as well as genes involved in direct defense responses are induced. Such coordinated regulation of source-sink relations and defense responses has been observed both for experimental model systems (Ehness et al., 1997) and various pathosystems (Berger et al., 2007). It has been shown that the coordinated photosynthesis, sink metabolism and defense responses may be independently regulated by both sugars and stress-related signals (Ehness et al., 1997). Since FDp infection results in an elevated level of carbohydrates, and some of the regulated genes, notably the highly induced SuSy (Godt et al., 1995), are known to be metabolically regulated by carbohydrates, it is not possible to discriminate whether the primary event is the generation of a metabolic sugar signal (Ruan et al., 2010; Tiessen and Padilla-Chacon, 2012) or a direct effect of FDp-related pathogen signal.

While the trends of grapevine responses here at the transcriptional level were similar to those previously shown for infection with another causal agent of grapevine yellows, BNp, the intensity of the changes were not always the same. These differences, which occurred especially in the expression profiles of genes that encode pathogenesis-related proteins, might contribute to the strong epidemic nature of FDp. The expression of genes encoding SWEET proteins was analyzed here for the first time in grapevine, and this study demonstrates a new pathogen association with SWEET17a from clade IV. FDp infection also triggers a change in amino-acid synthesis and in the metabolism of ascorbate and phenylpropanoids. With a new experimental platform for assay of the key enzymes of primary carbohydrate metabolism that has been adapted for grapevine leaves, we have substantiated the results of the analysis of carbohydrates. Particularly, we showed a prominent role of SuSy in FDp-grapevine interaction. Thus this study demonstrates that the various scales of spatial and temporal dynamics of physiological responses need to be integrated into a multidimensional phenomics approach for physiological fingerprinting at the cellular and tissue level to complement molecular markers (Großkinsky et al., 2015). The present study also revealed expression of *VvDMR6* early in the growing season, before the development of symptoms of FDp infection, to be potentially applied to early diagnosis of grapevine yellows.

## Author contributions

NP participated in the design of the study, collected the samples, carried out most of the experiments, and helped to write the manuscript; EC adapted enzyme assays to grapevine, tested the activities in BNp-infected grapevines, and wrote related sections of the manuscript; TR established the system for testing enzyme activities and helped to write the manuscript; KG coordinated the experiments that involved real-time PCR; MC and MV analyzed the SWEET genes/proteins; LF and WW supervised the metabolome and data analyses and interpretation. MD designed the study, coordinated it, and drafted and completed the manuscript. All of the authors have read and approved the final manuscript.

### Conflict of interest statement

The authors declare that the research was conducted in the absence of any commercial or financial relationships that could be construed as a potential conflict of interest.
