# Supplementary material for: Metabolic Consequences of Infection of Grapevine (Vitis vinifera L.) cv. “Modra frankinja” with Flavescence Dorée Phytoplasma
Source: Front Plant Sci. 2016 May 23;7:711. doi: 10.3389/fpls.2016.00711 (PMC4876132; doi:10.3389/fpls.2016.00711)
Supplement: Supplementary file 1 [file Table1.PDF]

Supplementary Material

Metabolic consequences of infection of grapevine (*Vitis vinifera* L.) cv. 'Modra frankinja' with flavescence dorée phytoplasma

Nina Prezelj, Elizabeth Covington, Thomas Roitsch, Kristina Gruden, Lena Fragner, Wolfram Weckwerth, Marko Chersicola, Maja Vodopivec, Marina Dermastia

Correspondence: marina.dermastia@nib.si

Supplementary Table S1. Sanitary status of grapevines cv. 'Modra frankinja' inside the quarantine net.

Sample	2010/ July 2011/August 2011	August 2010/August 2011	August 2010
	qPCR: FDp	qPCR: BNp	ELISA: Viruses
8/2	neg./ neg./neg.	neg./neg.	neg.
8/3	neg./ neg./neg.	neg./neg.	neg.
8/4	pos./ pos./neg.	neg./neg.	neg.
8/5	pos./pos./pos.	neg./neg.	neg.
8/6	neg./ neg./neg.	neg./neg.	neg.
8/7	pos./pos./ neg.	neg./neg.	neg.
8/10	pos./pos./pos.	neg./neg.	GFkV
8/11	neg./ neg./neg.	neg./neg.	NA
8/12	neg./ neg./neg.	neg./neg.	neg.
8/13	neg./ neg./neg.	neg./neg.	neg.

neg., negative
pos., positive
GFkV, *Grapevine fleckvirus*
NA, not applicable
