# Supplementary material for: Metabolic Consequences of Infection of Grapevine (Vitis vinifera L.) cv. “Modra frankinja” with Flavescence Dorée Phytoplasma
Source: Front Plant Sci. 2016 May 23;7:711. doi: 10.3389/fpls.2016.00711 (PMC4876132; doi:10.3389/fpls.2016.00711)
Supplement: Supplementary file 2 [file Table2.pdf]

Supplementary Material

Metabolic consequences of infection of grapevine (*Vitis vinifera* L.) cv. 'Modra frankinja' with flavescence dorée phytoplasma

Nina Prezelj, Elizabeth Covington, Thomas Roitsch, Kristina Gruden, Lena Fragner, Wolfram Weckwerth, Marko Chersicola, Maja Vodopivec, Marina Dermastia

Correspondence: marina.dermastia@nib.si

Supplementary Table S2. Names of predicted genes encoding SWEET proteins in the grapevine genome, with their corresponding accession numbers.

Clade	Name	GenBank ID	Grimplet ID*
Clade I	*Vv_SWEET1*	XP_002265836.1	VIT_18s0001g15330
	Vv_SWEET2a	XP_002269484.1	VIT_10s0003g02190
	Vv_SWEET2b	XP_002285636.1	VIT_19s0014g00280
	Vv_SWEET3	XP_002267886.1	VIT_16s0050g02540
Clade II	*Vv_SWEET4*	XP_002263697.1	VIT_02s0025g02080
	Vv_SWEET5a	XP_002283068.1	VIT_17s0000g08110
	Vv_SWEET5b	XP_002279850.1	VIT_17s0000g08130
	Vv_SWEET6	XP_002274582.1	VIT_14s0066g01420
Clade III	*Vv_SWEET10*	XP_002284244.1	VIT_17s0000g00830
	Vv_SWEET14a	XP_002270131.1	VIT_07s0104g01340
	Vv_SWEET14b	XP_002280599.1	VIT_17s0000g00820
	Vv_SWEET15	XP_002264875.1	VIT_01s0146g00260
Clade IV	*Vv_SWEET16*	XP_002279031.1	VIT_14s0060g01910
	Vv_SWEET17a	XP_002269234.1	VIT_05s0077g02260
	Vv_SWEET17b	XP_002278982.1	VIT_14s0060g01890
	Vv_SWEET17c	CBI39543.3	VIT_14s0060g01880

* ID follows the grapevine gene nomenclature system by Grimplet *et al*. 2014.

Grimplet, J., Adam-Blondon, A.F., Bert, P. F., Bitz, O., Cantu, D., Davies, C., et al. (2014). The grapevine gene nomenclature system. BMC Genomics 15:1077. doi: 10.1186/1471-2164-15-1077
