# Supplementary material for: Metabolic Consequences of Infection of Grapevine (Vitis vinifera L.) cv. “Modra frankinja” with Flavescence Dorée Phytoplasma
Source: Front Plant Sci. 2016 May 23;7:711. doi: 10.3389/fpls.2016.00711 (PMC4876132; doi:10.3389/fpls.2016.00711)
Supplement: Supplementary file 3 [file Table3.PDF]

Supplementary Material

Article Title

Metabolic consequences of infection of grapevine (*Vitis vinifera* L.) cv. 'Modra frankinja' with flavescence dorée phytoplasma

Nina Prezelj. Elizabeth Covington. Thomas Roitsch. Kristina Gruden. Lena Fragner. Wolfram Weckwerth. Marko Chersicola. Maja Vodopivec. Marina Dermastia
Correspondence: marina.dermastia@nib.si

Supplementary Table S3. Annotated and putatively annotated compounds in leaf-vein samples of cv. 'Modra frankinja'. The ions used for quantification of the compounds are denoted as *m/z*. tr. retention time; RI. retention index; TMS. trimethylsilylation; MeOx. methoximation.

Parameters used for peak detection and deconvolution in AMDIS		
Identification:	Type of analyses	Use Retention Indes Data
	RI window	5 + 0.01 RI
	Match factor penalties:	Level: very strong
		Maximum penalty: 20
		No RI in Library: 20
Instrument:	m/z range	auto
	Treshold	Off
	Scan direction	None
	Instrument type	quadrupole
	Data file format	Xcalibur Raw Files
Deconvolution	Component width	12

Omit m/z	207, 221, 281, 355
Adjacent peak substraction	One
Resolution	Low
Sensitivity	Medium
Shape requirements	Low

Analyte*	Quant *m/z*	RT	RI	Sample	ΔRI (customized gmd)	Match/Reversed Match (customized gmd)	ΔRI (Mosys in-house)	Match/Reversed Match (Mosys in-house)	Database	MSI level of identification confidence**
Pyruvate (1MeOX) (1TMS)	174	9.34	1067	8/4	29.6	871/871	18.9	869/869	in-house	1
Glycolate (2TMS)	177	9.9	1086	8/13	23.6	821/821			gmd	2
Valine (1TMS)	72	10.4	1101	8/4	19.1	907/909			gmd	2
Alanine (2TMS)	116	10.8	1113.1	8/13	24.7	852/852	6.1	849/849	in-house	1
Glycine (2TMS)	102	11.46	1134.6	8/4	25.53	868/868	4	851/921	in-house	1
Leucine (1TMS)	86	12.45	1165.8	8/4	14.74	862/862			gmd	2
Proline (1TMS)	70	12.89	1180.1	8/4	4.1	889/894			gmd	2
Malonic acid (2TMS)	233	13.9	1215	8/4	17.84	807/807			gmd	2
Valine (2TMS)	144	14.16	1225.9	8/4	18.8	866/866	-1.2	806/806	in-house	1
Serine (2TMS)	219	15.21	1268.7	8/4	16.2	893/918			gmd	2
Ethanolamine (3TMS)	174	15.43	1277.5	8/4	17.4	875/883			gmd	2
Leucine (2TMS)	158	15.62	1285.6	8/4	21.17	965/965	-1.2	890/890	in-house	1
Phosphoric acid (3TMS)	283	15.77	1291.2	8/4	28.78	801/832			gmd	2
Glycerol (3TMS)	218	15.81	1292.7	8/4	30.4	887/892			gmd	2
Proline (2TMS)	142	16.15	1306.5	8/13	10.73	775/886	-2.7	826/967	in-house	1
Threonine (2TMS)	130	16.18	1308	8/13	17.55	765/765			gmd	2
Maleate (2TMS)	245	16.4	1316	8/13	15.1	902/915			gmd	2
Glycine (3TMS)	248	16.43	1317.8	8/4	15.77	863/875	-2.3	800/800	in-house	1
Succinate (2TMS)	247	16.6	1324	8/4	13.15	924/931	-3.5	923/923	in-house	1

Glycerate (3TMS)	117	17.18	1348.4	8/4	28.4	882/897			gmd	2
Fumarate (2TMS)	245	17.34	1355	8/4	8.2	913/928	-3.1	913/913	in-house	1
Alanine (3TMS)	188	17.65	1367.6	8/4	11	907/924	-4.2	894/928	in-house	1
Serine (3TMS)	278	17.85	1375.2	8/4	22.3	930/943	-3.2	900/905	in-house	1
Threonic acid-1.4-lactone (2TMS)	247	18	1382.7	8/4	10.1	905/921			gmd	2
2-Methyl-1.3-butanediol (2TMS)	306	18.2	1390	8/4	-4.3	848/848			gmd	2
Threonine (3TMS)	218	18.47	1400.3	8/4	23.1	907/923	-1.8	959/981	in-house	1
2.4-Dihydroxy-Butanoic acid (3TMS)	103	19	1426.8	8/4	21.88	824/866			gmd	2
Aspartate (2TMS)	160	19.09	1430.6	8/4	8.6	903/917			gmd	2
b-Alanine (3TMS)	248	19.2	1435.2	8/4	10.81	841/841			gmd	2
Citramalate (3TMS)	247	20.3	1488	8/4	23.7	848/866			gmd	2
Malate (3TMS)	335	20.7	1507.8	8/13	28.9	908/925	-1.4	895/901	in-house	1
Salicylate (2TMS)	267	20.97	1520.9	8/4	12.78	812/833			gmd	2
Pyroglutamate (2TMS)	258	21.22	1531.6	8/2	9.88	895/914			gmd	2
Aspartate (3TMS)	218	21.3	1536.9	8/4	25.9	875/893	-2.4	848/850	in-house	1
Glutamate (2TMS)	158	21.36	1539.8	8/4	11.6	877/893			gmd	2
Phenylalanine (1TMS)	120	21.56	1549.3	8/10	-6.75	708/877			gmd	2
Erythronic acid (4TMS)	292	21.92	1566.5	8/10	37.55	891/925			gmd	2
Threonate (4TMS)	319	22.31	1585.4	8/13	39.46	779/801	-3.1	899/903	in-house	1
2-Oxoglutarate (2TMS)	288	22.4	1589.8	8/4	16.95	825/833	-2.7	827/839	in-house	1
Glutamate (3TMS)	128	23.24	1634	8/4	19.4	897/931			gmd	2
Phenylalanine (2TMS)	192	23.27	1635.7	8/13	6.2	838/854	0.1	852/859	in-house	1
Aldopentose (1MeOx) (4TMS) 1	103	24	1678							3
Aldopentose (1MeOx) (4TMS) 2	103	24.2	1688							3
Aldopentose (1MeOx) (4TMS) 3	103	24.3	1694							3
Ketopentose (1MeOx)	263	24.6	1708							3

(4TMS)										
Pentose alcohol (5TMS)1	307	25.2	1738							3
Pentose alcohol (5TMS) 2	189	25.5	1756							3
Ribonic acid (5TMS)	333	25.95	1782.4	8/4	31.67	786/816			gmd	2
Glutamine (3TMS)	156	26.05	1787.3	8/13	20.5	904/932	-3.7	715/768	in-house	1
Lyxonic acid (5TMS)	333	26.2	1796	8/4	36.1	897/931			gmd	2
Arabinonic acid (5TMS) (P)	333	26.35	1804.4	8/4	39.56	780/873			gmd	2
Shikimate (4TMS)	372	26.87	1835.9	8/4	41.27	818/865			gmd	2
Citrate (4TMS)	273	27.04	1845.5	8/4	40.8	910/921	-2.7	811/811	in-house	1
Isocitrate (4TMS)	245	27.07	1847.6	8/2	42.2	775/793			gmd	2
Dehydroascorbate dimer (2MeOx)	157	27.5	1872	8/13	32.42	844/907			gmd	2
Quinate (5TMS)	255	27.85	1894.3	8/13	51.7	848/905			gmd	2
Fructose (1MEOX) (5TMS) MP	189	28.1	1910	8/13	56.1	897/926	-3.3	923/927	in-house	1
Pentahydroxyhexanoic acid-1.4-lactone (4TMS) P	361	28.2	1915.7						gmd	3
Galactose or Mannose (1MeOx) (5TMS)	318	28.4	1929						gmd	3
Glucose (1MeOx) (5TMS) BP	233	28.8	1953	8/13	72.6	857/893	-0.7	901/903	in-house	1
Ascorbate (4TMS)	345	29.2	1976.1	8/13	38.24	830/852	-0.2	790/825	in-house	1
Pentahydroxyhexanoic acid (6TMS) 2	292	30	2035			871/928				3
Pentahydroxyhexanoic acid (6TMS)1	333	30.1	2030			856/918				3
Palmitic acid (1TMS)	313	30.3	2045.9	8/13	-0.3	814/867			gmd	2
Pentahydroxyhexanoic acid (6TMS) 3	277	30.5	2056			811/849				3
Pentahydroxyhexanoic	333	30.9	2083			793/808				3

acid (6TMS) 4										
Caffeate (3TMS) (P)	396	31.84	2148	8/4	12.45	836/845			gmd	2
Stearic acid (1TMS)	341	33.2	2242	8/4	-1.7	814/873			gmd	2
Fructose-6-P (1MeOx) (6TMS) (P)	315	34.88	2359.1	8/4	66.62	807/849			gmd	2
Hexose-6-P (1MeOx) (6TMS)	315	35	2371	8/4	76.05					3
Hexose-6-P (1MeOx) (6TMS)	315	35.27	2388.2	8/4	80.86					3
Salicylic acid-glucopyranoside (5TMS) (P)	267	38.3	2621	8/4	52.97	866/923			gmd	2
Sucrose (8TMS)	331	39.37	2711.8	8/4	88.93	674/726	1.8	833/856	in-house	1
Saccharide 1	204	39.5	2722							3
Saccharide 2	204	39.6	2732							3
Saccharide 3	204	39.7	2743							3
Saccharide 4	261	39.8	2751							3
Saccharide 5	361	40.1	2773							3
Saccharide 6	204	40.4	2797							3
Saccharide 7	361	40.5	2806							3
Saccharide 8	204	40.7	2822							3
Saccharide 9	361	40.9	2838							3
Epicatechin (5TMS) (P)	355	41.48	2893	8/10	29.45	861/878			gmd	2
Catechin (5TMS) (P)	355	41.76	2918.5	8/4	52.94	874/914			gmd	2
Flavonoid	559	43.8	3106							3

*Analytes are given as trimethylsilyl (TMS) and/or methoxime (MeOx) derivatives
**MSI levels according to Metabolomics Standard Initiative (MSI) (Sumner et al. 2007)
Level 1, Identified metabolites
Level 2, Putatively annotated compounds
Level 3, Putatively characterised compound classes
Level 4, Unknown compounds
