# Supplementary material for: Metabolic Consequences of Infection of Grapevine (Vitis vinifera L.) cv. “Modra frankinja” with Flavescence Dorée Phytoplasma
Source: Front Plant Sci. 2016 May 23;7:711. doi: 10.3389/fpls.2016.00711 (PMC4876132; doi:10.3389/fpls.2016.00711)
Supplement: Supplementary file 4 [file Table4.PDF]

Supplementary Material

Metabolic consequences of infection of grapevine (*Vitis vinifera* L.) cv. 'Modra frankinja' with flavescence dorée phytoplasma

Nina Prezelj, Elizabeth Covington, Thomas Roitsch, Kristina Gruden, Lena Fragner, Wolfram Weckwerth, Marko Chersicola, Maja Vodopivec, Marina Dermastia

Correspondence: marina.dermastia@nib.si

Supplementary Table S4. Enzyme activity from whole-leaf samples collected in August 2011. S.e.m. is the standard error of the mean for triplicate samples. The measured enzyme activities of AGPase, VacINV and cwINW in August 2011 were in general higher than in August 2010, but the ratio between uninfected and infected samples remained similar.

	**AGPase**		**SUSY**		**nINV**		**vacINV**		**cwINV**	
**Sample**	Specific activity (nkat/g FW)	s.e.m.	Specific activity (nkat/g FW)	s.e.m.	Specific activity (nkat/g FW)	s.e.m.	Specific activity (nkat/g FW)	s.e.m.	Specific activity (nkat/g FW)	s.e.m.
8/2	2.72	0.06	0.09	0.006	0.56	0.01	5.2	0.08	4.05	0.14
8/3	3.49	0.06	0.15	0.008	0.54	0.01	3.97	0.15	2.63	0.08
8/4	3.5	0.09	0.18	0.013	0.87	0.03	7.34	0.12	7.13	0.08
8/5	3.48	0.18	0.59	0.024	0.94	0.07	8.66	0.23	4.2	0.11
8/7	3.69	0.03	0.09	0.031	0.62	0.01	6.52	0.09	5.17	0.14
8/10	3.6	0.03	0.8	0.036	0.83	0.01	9.1	0.17	6.19	0.16
8/12	9.19	0.15	0.13	0.018	0.59	0.01	3.78	0.11	3.55	0.13
