# Supplementary material for: Metabolic Consequences of Infection of Grapevine (Vitis vinifera L.) cv. “Modra frankinja” with Flavescence Dorée Phytoplasma
Source: Front Plant Sci. 2016 May 23;7:711. doi: 10.3389/fpls.2016.00711 (PMC4876132; doi:10.3389/fpls.2016.00711)
Supplement: Supplementary file 6 [file Image2.PDF]

## *Supplementary Material*

# Metabolic consequences of infection of grapevine (*Vitis vinifera* L.) cv. 'Modra frankinja' with flavescence dorée phytoplasma

**Nina Prezelj, Elizabeth Covington, Thomas Roitsch, Kristina Gruden, Lena Fragner, Wolfram Weckwerth, Marko Chersicola, Maja Vodopivec, Marina Dermastia**

**Correspondence:** marina.dermastia@nib.si

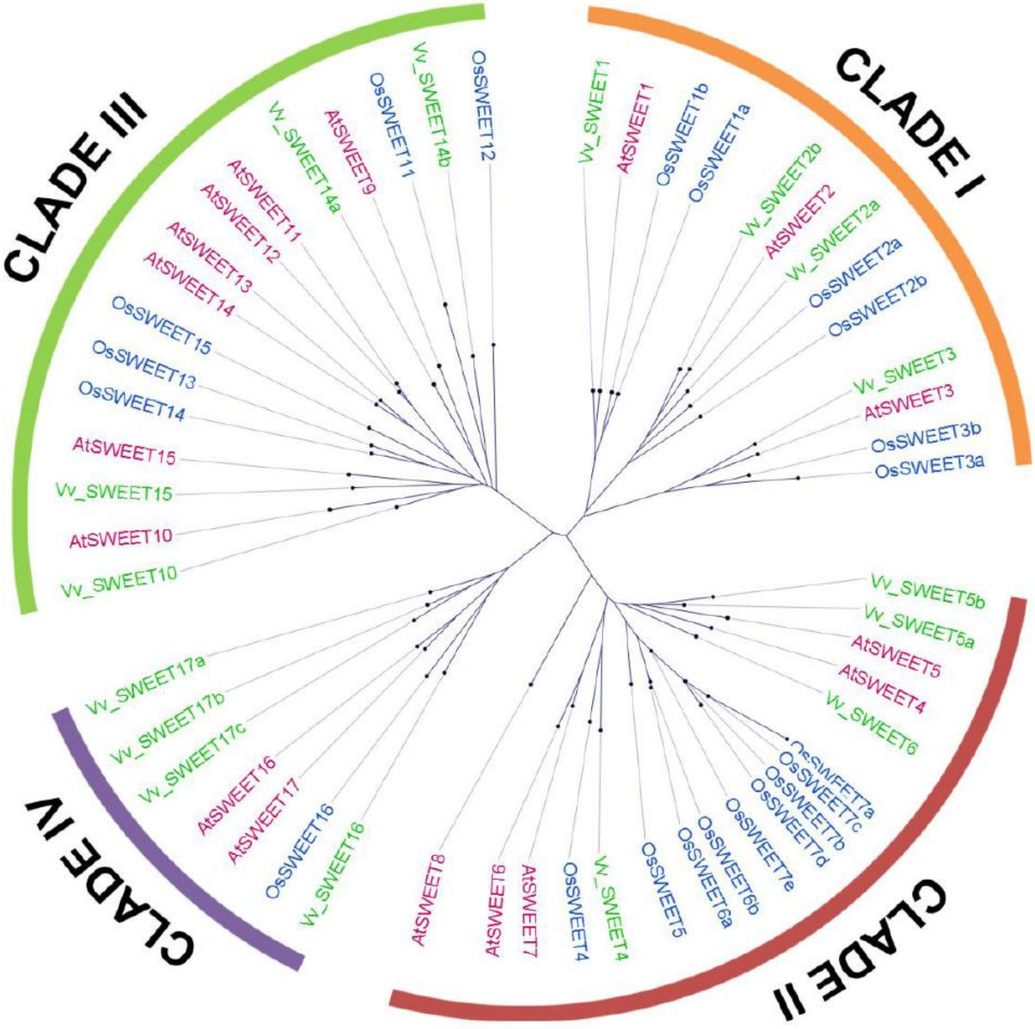


**Supplementary Figure S2.** Phylogenetic tree constructed from alignment of grapevine, rice and *Arabidopsis* SWEET protein sequences. They are classified into four clades. Green, grapevine sequences; blue, rice sequences; red, *Arabidopsis* sequences.
